# Segmental duplication as one of the driving forces underlying the diversity of the human immunoglobulin heavy chain variable gene region

**DOI:** 10.1186/1471-2164-12-78

**Published:** 2011-01-27

**Authors:** Sreemanta Pramanik, Xiangfeng Cui, Hui-Yun Wang, Nyam-Osor Chimge, Guohong Hu, Li Shen, Richeng Gao, Honghua Li

**Affiliations:** 1 Department of Molecular Genetics, Microbiology, and Immunology, University of Medicine and Dentistry of New Jersey-Robert Wood Johnson Medical School, Piscataway, NJ 08854, USA; 2 State Key Laboratory of Oncology in South China, Cancer Center, Sun Yat-Sen University, 651 Dongfeng East Road, Guangzhou 510060, China; 3 Environmental Health Division, National Environmental Engineering Research Institute, Nagpur- 440 020, India

## Abstract

**Background:**

Segmental duplication and deletion were implicated for a region containing the human immunoglobulin heavy chain variable (IGHV) gene segments, 1.9III/hv3005 (possible allelic variants of IGHV3-30) and hv3019b9 (a possible allelic variant of IGHV3-33). However, very little is known about the ranges of the duplication and the polymorphic region. This is mainly because of the difficulty associated with distinguishing between allelic and paralogous sequences in the IGHV region containing extensive repetitive sequences. Inability to separate the two parental haploid genomes in the subjects is another serious barrier. To address these issues, unique DNA sequence tags evenly distributed within and flanking the duplicated region implicated by the previous studies were selected. The selected tags in single sperm from six unrelated healthy donors were amplified by multiplex PCR followed by microarray detection. In this way, individual haplotypes of different parental origins in the sperm donors could be analyzed separately and precisely. The identified polymorphic region was further analyzed at the nucleotide sequence level using sequences from the three human genomic sequence assemblies in the database.

**Results:**

A large polymorphic region was identified using the selected sequence tags. Four of the 12 haplotypes were shown to contain consecutively undetectable tags spanning in a variable range. Detailed analysis of sequences from the genomic sequence assemblies revealed two large duplicate sequence blocks of 24,696 bp and 24,387 bp, respectively, and an incomplete copy of 961 bp in this region. It contains up to 13 IGHV gene segments depending on haplotypes. A polymorphic region was found to be located within the duplicated blocks. The variants of this polymorphism unusually diverged at the nucleotide sequence level and in IGHV gene segment number, composition and organization, indicating a limited selection pressure in general. However, the divergence level within the gene segments is significantly different from that in the intergenic regions indicating that these regions may have been subject to different selection pressures and that the IGHV gene segments in this region are functionally important.

**Conclusions:**

Non-reciprocal genetic rearrangements associated with large duplicate sequence blocks could substantially contribute to the IGHV region diversity. Since the resulting polymorphisms may affect the number, composition and organization of the gene segments in this region, it may have significant impact on the function of the IGHV gene segment repertoire, antibody diversity, and therefore, the immune system. Because one of the gene segments, 3-30 (1.9III), is associated with autoimmune diseases, it could be of diagnostic significance to learn about the variants in the haplotypes by using the multiplex haplotype analysis system used in the present study with DNA sequence tags specific for the variants of all gene segments in this region.

## Background

The human immunoglobulin heavy chain variable region (IGHV) is highly polymorphic [reviewed in [1]]. It is believed that no chromosomes share the same set of IGHV gene segments in the human population. However, the extent of the polymorphisms, their impact on antibody diversity and the mechanisms underlying the formation of these polymorphic regions remain unclear as studying polymorphisms in this region is a daunting task. The high degree of sequence identity among the repetitive sequences makes it extremely difficult to distinguish between allelic and paralogous sequences. Because the region is highly polymorphic, it is necessary to separate individual allelic variants or haplotypes for study of this region while the commonly available and analyzable samples are usually diploid. To overcome these barriers, we have developed a suite of innovative techniques. By using single sperm cells, the haplotypes in the polymorphic regions can be analyzed individually and precisely. Since paralogous sequences are present in the same sperm and allelic sequences always segregate into different gametes, these sequences can be readily distinguished in this way. Our high-throughput multiplex DNA sequence detection technique can detect a large number of genetic markers and DNA sequence tags in single haploid cells [2-4]. These techniques allowed us to indentify several polymorphic regions in the IGHV gene complex ([5-9], Pramanik et al., manuscript in preparation).

One of these polymorphic regions, designated as Region II, identified in our earlier studies is of particular interest. Region II was initially noticed when we mapped the gene segments in IGHV1 and IGHV4 families in two haplotypes [7]. Interestingly, all IGHV1 and IGHV4 gene segments were not detectable in a large block between IGHV gene segments, 4-4 and 4-31 in one haplotype, and three gene segments between 1-24 and 4-31 were missing in the other. Later, this region was detected again by Chimge et al. [5]. Surprisingly, in that study, only two gene segments (3-29P and 4-28.1P) were undetectable in one of the ten haplotypes in this region. Segmental duplication and deletion in this region were implicated for gene segments 1.9III/hv3005 (possible allelic variants of IGHV3-30) and hv3019b9 (a possible allelic variant of IGHV3-33) [10-14]. Later, additional gene segments DP46 and two copies of DP49 (possible allelic variants of IGHV3-30), and DP65 and DP78 (possible allelic variants of IGHV4-31) were shown to be located within the duplicated region [15]. All these gene segments fall into Region II detected in the present studies. Results from the above studies raise several questions: (1) why is the polymorphic region detected with different sizes and different frequencies in these studies; (2) if the segmental duplication underlies the polymorphism, what is the size of the duplicated region, and how are the duplicated blocks organized; (3) how many gene segments are affected by the duplication/deletion; (4) how much have the gene segment variants diverged; and (5) what is the biological impact of this polymorphism. The present study was designed to answer these questions.

## Results

**Figure 1:**
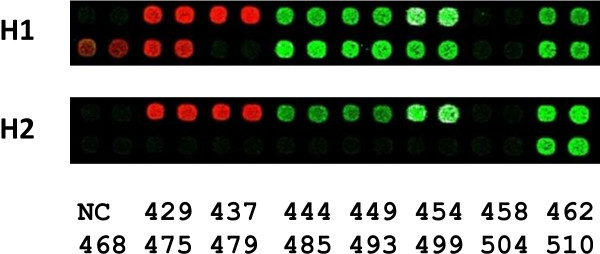
**Microarray images for sperm with two different parental haplotypes, H1 and H2, from Donor AB027**. Each probe was printed twice as adjacent spots in the same row. The corresponding tag names for the array spots are indicated on the right as numbers with their prefix "VHS" left out (Note, each number represents two adjacent spots). NC: negative controls with a probe for an undetectable sequence.

DNA sequence tags (≤ 225 bp, n = 17), with an average spacing of ~5 kb were selected from the previously described polymorphic region, Region II, including certain flanking sequences on both sides, spanning 89,839 bp in the GRCh37 Human Genome Reference Assembly http://www.ncbi.nlm.nih.gov/genome/guide/human/release_notes.html (Table 1). Genotypes (detectable/undetectable) of the tags were determined by analyzing 49 to 60 single sperm samples from each of six sperm donors after multiplex PCR amplification and microarray detection. Microarray images for two single sperm samples of different parental origins from Donor AB027 are shown in Figure 1. Table 2 summarizes the results.

**Table 1:** Sequences of primers and probes

**Tag**			**Primer (5' to 3')**			
				
	**Location**					**Probe (5' to 3')**
			
**Name**	**from**	**to**	**Forward**	**Reverse**	**Nested**	
				
VHS429	428971	429130	ATATATACAAGATTAGTCCCACAATA	ATACCAGCTCTACTATATATGAAAGTC	TATGCAAGCATTAGATACCCATA	AAACCATGCTGTCGCAGGGTC
VHS437	437246	437398	CACAAAATGCAGATTCACACTC	AGCCTGTTCTAGTCATGGAATCTC	GAAAAGTCCTCAACTGTGGTCA	CCAGGAACGCCTGAGATTCCT
VHS444	444731	444867	CACCCCTTCACAACTGGGGAT	TGGTAGCGCTGTGAGGTGATA	TGGGGCAGGACACAAATCCAA	TACCTGACCACCATGCGTATT
VHS449	449260	449427	GAACAGACAACCTGTGAATGGT	ATTTCTGTTGAACACTTGTATGTCT	AAATGTGCAGTCTATTCATCAAAT	TATCTCGTTTTTGCTCACTCT
VHS454	454027	454155	TATTACCAGGAGACCAAAATCTC	AAAGGAAGGTTGCTGGTAAGG	ATACAGACAAACAGGCGAAGG	GATTGCCTTTCGCCACATAGA
VHS458	458073	458214	CGAAATATAATTCTTGCTCTTCTG	CAGGAGAATTATGAACATTGAGAG	AGCACATCCTAGCATCCCTGT	CATCGGCGTGTTTACATCTAC
VHS462	462778	462952	CGTTAGCTCTGTGAAAGCAGC	TGATTTGTTCCCTTAGTTTCTGG	CAGGTTTACTGTTAAGGAGGTCA	TGGCTACTCAGCGTGCAAATG
VHS468	468656	468843	TTAGCATCTCCTATTTGAGTAGATTT	TCTAGATATTCAGGAGGCTAATACA	TGTTCCACAGCTTAAAGTACAGTT	TGAGTCCGGAGCTCAAAATTA
VHS475	475730	475892	TGTAGTTTCCTTTATTGTGGTGC	AATTCTTCCCAACAGAAAAGTG	TTACTGGTAATACTATCATGGTAGTC	AGGTAGTCTGCCATACACCTT
VHS479	479662	479810	GAAGCAAATTGATTAGTGTGCAG	CAGCTGAATCGCTTTTGGTC	TCTGCTTTGCCTGCTATTCAT	TGAGAAACACACGGGTCTTAT
VHS485	485677	485819	GCTGTCTCCTAAGTAAGTCACAGG	AGGGATTCAGTGCAAATTGAG	ACACACCAACATCACCAACAT	TCTATAGTTCGTTCGAGGAAGT
VHS493	493051	493239	GAGTGAGCCTTAAGTAATCAACAG	CCCATCCCCTCCTTCTTTCTC	TGACAACTTATTCTAGGTAGCAAGA	CTGGGTCTTGTCCATCGATAC
VHS499	499270	499449	CTGAAGCTGCTTAATCACCGT	TGTGTATTGATTTGACAAAACACTA	TCTGCTCTGATGGAATCAGGG	ATCGGTTGACTCTATGTTAGG
VHS504	504031	504171	TGTGGGTTGTTCTCACCATAATA	ACAACTGGATGCACCTCCATA	CCACCAGACACCCTCCAATAC	CACTCCGACACATTATAAGAGA
VHS510	509963	510127	GTCGTGTATCTCACTTTCCACTA	TTTAGTGGGATTTTAGAGAGTACAA	GATGGAGTCCTGATCCCTGCC	TTTCTGTGACCGTGTGTCACT
VHS514	514084	514235	GAGTGTGGACTGATCCATTGC	ATACCATGTGCTGCTGACACC	ATAGAATCCTTGCTTGGGGTCT	CTAAACTGGCCGTAGGAAAGC
VHS518	518664	518809	CAAACCTGTCAGGGCACTTAG	TGACAGTAAACCAGCCTCTCAT	TCCAGGAAGACTCAAGACCAC	CCCCAAGTGGTCGTGAGTCCC

*Oligos are named using the corresponding tag names followed by a letter of F (forward primer), R (reverse primer), N (nested primer), or B (probe).

**From the 5' end of the IGHV region.

**Table 2:** Genotypes of the DNA sequence tags

			Donor and Haplotypes**											
			
Repeat	Tag(s)*		#002		#12		AB005		AB027		AC09		D18	
							
	Name(s)	No.	H1	H2	H1	H2	H1	H2	H1	H2	H1	H2	H1	H2
	VHS429 to 454	5	+	+	+	+	+	+	+	+	+	+	+	+
	VHS458	1	ud	ud	ud	ud	ud	ud	ud	ud	ud	ud	ud	ud

I	VHS462	1	+	+	+	+	+	+	+	+	+	+	+	+
	VHS468	1	+	+	ud	ud	+	+	+	ud	+	+	+	+
	VHS475	1	+	+	ud	ud	+	+	+	ud	+	+	ud	+
	VHS479	1	ud	ud	ud	ud	ud	ud	ud	ud	ud	ud	ud	ud

II	VHS485	1	+	+	ud	+	+	+	+	-	+	+	+	+
	VHS493	1	+	+	ud	+	+	+	+	-	+	+	+	+
	VHS499	1	+	+	ud	+	+	+	+	-	+	+	+	+
	VHS504	1	ud	ud	ud	ud	ud	ud	ud	ud	ud	ud	ud	ud

	VHS510	1	+	+	ud	+	+	+	+	+	+	+	+	+
	VHS514 & 518	2	+	+	+	+	+	+	+	+	+	+	+	+

*The ranges of consecutive non-polymorphic tags are indicated by the two ending tags and the number (No.) of tags in the respective range.

**"+": detectable, and "ud": undetectable."

### Haplotype patterns of the polymorphic tags

As shown in Table 2, the polymorphic region is flanked by five consecutive non-polymorphic tags (VHS429 to 454) on one side and two (VHS514 and 518) on the other. Two undetectable tags, VHS458 and 504, are immediately adjacent to this region. The region is divided into two nearly equal portions by VHS479. Four of the 12 haplotypes (shown as columns) were shown to have two or more consecutively undetectable tags. Undetectable tags in Haplotype 1 of Donor #12 are distributed consecutively in the entire polymorphic region. Region containing undetectable tags in Haplotype 1 in Donor D18 is the smallest which contains only two tags.

### Large duplicate blocks identified by sequence analysis

To learn about the cause and detailed content of this polymorphic region, sequences of the IGHV region from the three human genomic sequence assemblies http://www.ncbi.nlm.nih.gov/genome/guide/human/release_notes.html in the database maintained by the National Center for Biotechnological Information (NCBI) were analyzed. The GRCh37 Assembly is an updated version from the international Human Genome Project; the HuRef Alternative Assembly represents a composite haploid version of a single individual's diploid genome sequence [16], and the Celera Alternative Assembly is an integration of genomic sequences of five human individuals [17]. Although quite a few human whole-genome have been sequenced by next generation sequencing, these sequences were generated from diploid DNA samples and assembled from short sequence reads using known sequences as references. The suitability of these sequences for study of highly polymorphic regions needs to be further demonstrated. Therefore, these sequences were not included in the comparison.

**Figure 2:**
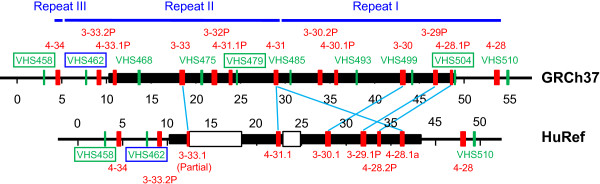
**Relative locations of IGHV gene segments (red vertical bars) and DNA sequence tags (green vertical bars) in the polymorphic portion of Region II**. Repeating blocks in the GRCh37 Assembly are shown as blue lines on the top. Polymorphic portion between the two assemblies, GRCh37 and HuRef, are represented by thick black strips. Gene segments with the closest sequences in the two assemblies are linked by light blue lines. Names of the undetectable tags are in green boxes and that for the non-polymorphic tag is in blue. Undetermined sequences are shown as hollow green rectangles. The right side of the region is IGHJ proximal.

We first analyzed the internal structure of Region II in the GRCh37 Assembly. A repeating sequence block was revealed between tags VHS458 and 510 (Figure 2). It has two complete copies that are 24,696 bp and 24,387 bp, respectively. A third copy is incomplete with only 961 bp. These copies correspond to the sequence from position 106,780,259 to 106,830,249 on chromosome 14 (NC_000014.8 in GRCh37). In all three genomic assemblies, the region starts with a sequence stretch of CAAGGATGTGAGGAAGTAGAACCACAGATAATAAAGAAAGAGGAGTCCTGGGGACAGCTG and ends with CTTTTGGGCTCACCCTGGGAGGTGTATGCTGGCTGTGCCCTCTGAGAACTCAGTTCTCTT. Each of the two complete copies contains six IGHV segments which are in the same order and share a sequence identity of 93% to 99% between the counterparts (Table 3). The only gene segment, 4-34, in the incomplete copy shares a sequence identity of 90% with the two counterparts in the complete copies.

**Table 3:** Difference between closely related gene segments

Difference	Name	Length	Name	Length	Difference	
					**bp**	**%**

	**GRCh37**		**HuRef**			
	4-28.1P	253	4-28.2P	255	2	0.78
	3-29P	458	3-29.1P	458	6	1.31
**Allelic**	3-30	454	3-30.1	454	1	0.22
	4-31	438	4-28.1a	438	19	4.34
	4-31	438	4-31.1	438	8	1.83
	3-33	454	3-33.1 (partial)	202	3	1.49
	Total			2245	39	1.74

	**Repeat I**		**Repeat II**			
	**4-28**	435	4-31	438	29	**6.62**
	4-28.1P	253	4-31.1P	255	12	4.71
	3-29P	458	3-32P	458	13	2.84
**Paralogous in GRCh37**	3-30	454	3-33	454	4	0.88
	4-30.1P	274	4-33.1	274	5	1.82
	3-30.2P	449	3-33.2P	449	8	1.78
	**Repeat I**		**Repeat III**			
	4-28	438	**4-34**	433	41	**9.36**
	**Repeat II**		**Repeat III**			
	4-31	438	**4-34**	433	43	**9.82**

	**Repeat I**		**Repeat I**			
	**4-28**	435	4-28.1a	438	28	**6.39**
	**Repeat I**		**Repeat II**			
	**4-28**	435	4-31.1	438	15	3.42
**Paralogous in HuRef**	4-28.1a	438	4-31.1	438	17	3.88
	3-30.1	454	3-33.1 (partial)	202	3	1.49
	**Repeat I**		**Repeat III**			
	4-28.1a	438	**4-34**	433	43	**9.93**
	**Repeat II**		**Repeat III**			
	4-31.1	438	**4-34**	433	41	**9.47**

### Divergence among the polymorphic variants

**Figure 3:**
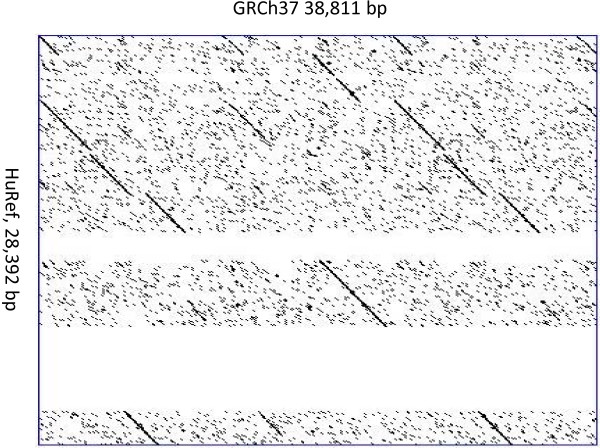
**Dot-matrix plot comparison between the polymorphic sequence variants in the GRCh37 and HuRef assemblies**. White spaces are undetermined sequences in HuRef. Window size, 9.

We further explored this duplicate region by comparing the sequences in the three genomic assemblies. Figure 3 shows an image of a comparison between the GRCh37 and HuRef Assemblies (Region II in the HuRef assembly corresponds to the sequence from 86,921,230 to 86,960,742 in AC_000146.1) using a dot matrix plotting program http://www.vivo.colostate.edu/molkit/dnadot/. As shown, a number of sequence blocks share a high degree of sequence identity between the two assemblies. However, discontinuity of these blocks indicates that extensive genetic shuffling has occurred in this region.

The divergence between the two assemblies is also reflected by the number, composition and organization of the gene segments. As shown in Figure 2, the variable portion between the two assemblies is 38.8 and 28.4 kb in the GRCh37 and HuRef assemblies, respectively. In this portion, the GRCh37 assembly contains ten IGHV gene segments, 4-28.1P, 3-29P, 3-30, 4-30.1P, 3-30.2P, 4-31, 4-31.1P, 3-32P, 3-33, and 4-33.1, while six gene segments are seen in the HuRef assembly. Because these six gene segments are all different from those in GRCh37, they are designated as IGHV4-28.1a, 4-28.2P, 3-29.1P, 3-30.1, 4-31.1 and 3-33.1. IGHV3-33.1 is a partial gene segment compared with the closest gene segment, IGHV3-33, in the GRCh37 assembly. Although the HuRef has two undetermined sequence blocks which may harbor more genes, the differences between the two assemblies are obvious: (1) 3-33.1 is the most 5' gene segment in HuRef, while GRCh37 has an additional gene segment, 4-33.1P; (2) 4-28.1a in HuRef is an inserted gene segment compared with GRCh37; and (3) based on the size of the smaller undetermined region (green rectangle on the right in Figure 2), HuRef may not have the counterparts gene segments for 4-30.1P and 3-30.2P found in the GRCh37 Assembly.

Back to the haplotype configurations in Table 2, it is obvious that the boundaries of the variable portion are consistent with those in the two sequence assemblies. This portion in all samples is flanked by two non-polymorphic tags, VHS462 and VHS510, with VHS510 in Haplotype 1 of Donor #12 being an exception. The variable portion of Haplotype 2 in Donor #12 and Haplotype 1 in Donor D18 involves only one repeating unit and that of Haplotype 1 in Donor #12 and Haplotype 1 in AB027 involves both complete copies. The three undetectable tags 458, 479 and 504 display a periodic pattern although VHS 458 is not located within the duplicated region.

At the nucleotide sequence level, the gene segments between the two assemblies are surprisingly diverged to a degree that is significantly higher than the genomic average rate of one base substitution for each 1,200 bp, or 0.08%. As shown in Table 3, the differences between the allelic counterparts in the two assemblies range from 0.22% to 4.34% or 2.6 to 52.1 bases when translated into the number of differences for each 1,200 bp, reflecting considerably higher mutagenic activities and limited selection pressure in this region. If the mutation rates are the same between the alleles and the paralogous copies, the allelic differences should be similar to the differences between the paralogous gene segments. Indeed, results from most gene segments in Table 3 support this notion. The allelic and paralogous differences between the gene segments are very close and there is no clear cut way to distinguish between these two types of sequences. This is a serious challenge for study of these regions, and also demonstrates the importance of using haploid material for study of this type of regions. Exceptions are two boundary gene segments, 4-28 and 4-34, which have identical alleles in difference assemblies and are much more different from their paralogous counterparts. This could be, at least in part, caused by the divergence of gene segments located within the duplicated region.

Sequence for the major portion of the polymorphic region (from 86,710,749 to 86,768,170 in AC_000057.1) in Celera Assembly remains undetermined. Sequence data is available for only three small fragments of 2176 bp, 1001 bp, and 132 bp which are closer to the sequence in Repeat II than that in Repeat I in GRCh37. Difference in these small regions between Celera and GRCh37, between Celera and HuRef, and between HuRef and GRCh37 is 2.92%, 3.19% and 3.60%, respectively compared with 0.08% for the genomic average, reflecting a high level of mutation rate in the intergenic regions. These rates are also significantly higher than the average allelic difference in the gene regions which is 1.74% (Table 3).

### Impact of sequence divergence on the function of IGHV gene segments

Functional paralogous counterparts of IGHV gene segments are found in the two complete copies and the incomplete copy of the duplicate block in the GRCh37 Assembly (Figure 2). These include two IGHV3 gene segments, 3-30 and 3-33, and three IGHV4 gene segments, 4-28, 4-31 and 4-34. Although the three IGHV4 gene segments have considerably diverged from each other (more than 9% difference between 4-34 and the other two and 6.62% between 4-28 and 4-31, Table 3), and four single-nucleotide differences are found between the two IGHV3 gene segments (Table 3). The exons of these gene segments all remain in Open Reading Frames (ORFs).

The HuRef assembly has two IGHV4 gene segments, 4-28 and 4-34, outside of the polymorphic region. These gene segments are identical to their allelic counterparts in GRCh37. Allelic counterparts for the remaining three functional gene segments in GRCh37 are all found in HuRef. In addition, HuRef has an extra gene segment, 4-28.1a which is mostly close to 4-31. As mentioned above, the allelic differences between the counterpart gene segments in the polymorphic region of the two assemblies range from 0.22% to 4.34% (Table 3). However, exons in all these four gene segments including 3-33.1 in HuRef are in ORFs. Only a partial sequence of 3-33.1 is known. Its exon 1 and the known portion of exon 2 are also in an ORF.

By contrast, the differences between paralogous IGHV gene segments within each complete duplicate block are much greater than those between the counterparts of the duplicate copies. As shown in Figure 2, each complete duplicate block contains three IGHV3 gene segments and three IGHV4 gene segments, one functional and two pseudogene segments for each family. Both IGHV3 pseudogene segments are in full length compared with the functional ones while the two IGHV4 pseudogene segments are truncated at about 40 bp upstream of exon 2. Within each duplicate block, sequence identity between the truncated pseudogene segments and the corresponding portions of the full length ones and between the full-length gene segments for each of the two families are under 80%, indicating that divergence among these gene segments occurred much earlier than the block duplication in this region.

### Sequence variation and tag detection

The hyper-variability in Region II may have significant impact on tag detection. Tags that are undetectable in a consecutive pattern may indicate structural alterations. As shown in Table 2 and Figure 2, the patterns of the undetectable tag clusters are consistent with the range of repeat units. Table 2 also shows that in all haplotypes, tags 458, 479 and 504 were undetectable while 462 was detectable. We first confirmed these experimental results independently by amplifying these tag sequences individually with 8 to 9 single sperm samples from donor AC09 followed by gel electrophoresis. All results obtained in this way were consistent with those obtained by multiplex PCR and microarray. This is understandable because the IGHV sequence that we used for tag selection was in the GRCh37 assembly and was from a Japanese genome by Matsuda et al [18] while the sperm donors used in the present study were all Caucasians. Tag 462 that was detectable in all individuals may be located in a region that is conserved between the two populations and tags that were undetectable may be affected by sequences polymorphic between Japanese and Caucasian. Since the donor of HuRef is a Caucasian [16], we compared the tag sequences between GRCh37 and HuRef for the above four tags. As expected, no difference was found between the two assemblies for tag 462 while the other three have multiple polymorphic bases in either primers, probes or both. For tag 458, a two-base substitution is located in the middle of the probe region and the template base for fluorescently labeled base incorporation is also polymorphic which alone makes the detection of the allele in HuRef impossible. In tag 479, two polymorphic bases affect the R primer and three are located in the probe region. For tag 504, two polymorphic bases are located in the middle of the probe region, and one is within and next to the 3' end of the F primer which may seriously affect the amplification efficiency.

## Discussion

### Why haploid genomes?

Evidence for extensive polymorphisms in the IGHV region has been observed in a large number of studies (reviewed in [1]). However, detailed analysis of polymorphic regions consisting of highly repetitive sequences represents a serious challenge. Of the gaps left by the Human Genome Project, 52% are regions containing repetitive sequences [19]. This is probably why Celera and HuRef assemblies have large blocks of undetermined sequences in the IGHV region. As shown in Table 3, in many cases the differences between allelic and paralogous sequences are very close. When they are present in the same diploid sample, no set measurement can be used to distinguish between these two types of sequences. Although the heterozygous state of a tag can be determined using a quantitative method, it would be difficult to learn the haplotype configurations of the polymorphic tags in the entire region. We unmasked the polymorphic regions and distinguished between allelic and paralogous sequences by analyzing individual haplotypes in single sperm cells. Using evenly spaced unique sequence tags of a high density, we identified polymorphic regions unambiguously. The haplotype configurations of the polymorphic tags could also be determined.

Three genomic sequence assemblies, i.e., GRCh37, HuRef and Celera, were used for sequence analysis of the polymorphic region. Although these assemblies were generated using diploid genomic DNA, they are composite haplotype sequences because individual DNA clones used for sequencing were derived from individual haplotypes. On the other hand, because of the presence of different variants, it is possible to have clones with significantly different sequences for a polymorphic region. Clones of different haplotype origins could be very confusing. The level of confusion may depend on the size of clones, the number and density of polymorphic sites and the overlapping size between adjacent clones. However, given the high density of polymorphic sites in Region II, the chance of constructing a contig composited from different haplotypes at sequence level would be very low. If the number of polymorphic sites in the overlapping regions is insufficient in a region for building up the haplotype contigs, the authors would treat the region as undetermined.

### Natural selection and gene function in Region II

Polymorphisms generated from non-reciprocal meiotic rearrangements in regions consisting of duplicated blocks may represent one of the major causes underlying the IGHV region diversity. As shown in Figure 3, the highly diverged sequence spacers between the conserved blocks indicate that complex genetic events may have occurred in this region so that these variants have considerably diverged from each other. As the field progresses rapidly, the gene-nursery role of low-copy repeats (LCRs) has become more and more evident. New genes may form through various nonreciprocal crossovers in the LCR regions resulting in duplication, deletion, inversion, translocation, and gene conversion/fusion/fission (reviewed in [20-22]). In some cases, gene number/structure changes may subject to greater selection pressure, while in other cases changes may not be affected or even favored by natural selection. In the latter cases, the LCR regions may display a complex polymorphic pattern. The scenario in Region II described in the present study likely belongs to the latter category. Although changes in this region involve many gene segments, it is difficult to imagine how much and how fast the selection pressure would affect the number and composition of the IGHV gene segments. Therefore, newly formed gene segments may have diverged from their original form so that no gene segment in the HuRef assembly is identical to those in the GRCh37 assembly in Region II. However, selection pressure is implicated in the gene regions as the intergenic difference ranged from 2.92% to 3.60% while the allelic difference is only 1.74%, indicating the functional importance of the gene segments in this region.

It seems surprising that none of the paralogous and the allelic counterparts in ORFs was inactivated although in many cases, differences between paralogous counterparts and those between allelic counterparts for the gene segments in ORFs are either greater than or comparable to the differences between their pseudogene counterparts (Table 3). A natural question would be why these gene segments remain in ORFs or whether the selection pressure has played a role in keeping their ORFs? The fact that among the 123 IGHV gene segments reported by Matsuda et al. [18], 79 (64%) are pseudogenes indicated that selection effect in the IGHV region is not evident in general.

Two types of genetic alterations may inactivate an IGHV gene segment, i.e., point or minor mutation and unequal meiotic crossover. By sequencing complete genomes of a human family of four members (parents and two children), Roach et al. [23] showed that the mutation rate in the human genome is 1.1 × 10^-8 ^bases/base/haploid genome/generation. Given that 18 of the 61 non-stop genetic codons can be changed into stop codons by changing a single base, the total length of the two exons in each IGHV gene segment is ~355 bp, and a human generation is 25 years, about 21.7 million years is needed to change an IGHV gene segment in an ORF into a pseudogene segment. Among the 79 IGHV pseudogenes, 26 were inactivated by point mutations or other minor changes, and 53 which are about twice of those caused by point and minor mutations were caused by major structural changes with the majority by truncations. Based on this rate, if both genetic alterations responsible for structural changes and point mutation are taken into consideration, 7.2 million years are need to inactivate an IGHV gene segment in an ORF. On the other hand, the difference between the two complete repeats in Region II is 4.36% (not including an Alu insertion in Repeat I), which needs 99.0 million years to achieve, 13.7 times longer than the time needed for inactivate a gene segment. One may argue that the mutation rate may be significantly higher in the IGHV region than other chromosomal regions. However, a generally higher rate for the IGHV region should not affect comparison between the gene regions and other sequence in the repeats.

The fact that gene segments in ORFs remain in ORFs after duplication indicates that selection pressure may have played an important role in keeping these gene segments functional. This hypothesis is further supported by the fact that there are only about eight IGHV4 gene segments in each haploid genome, three (37.5%), i.e., 4-28, 4-31 and 4-34, are located in the duplicated region. Therefore, functionality of these genes may have significant impact on resistance to certain severe diseases. Other than the segmental duplication reported in our present study, Walter et al. [15] also observed duplication of 3-30 and 4-31 segments, which may enhance the carriers' immune capacity. However, some of these gene segments may be related to the susceptibility of diseases. For example, the unchanged germline form of 1.9III, an allelic variant of IGHV3-30, may encode an autoantibody [24-26].

To learn the causes for gene segment truncation, we analyzed the flanking sequences for the four truncated gene segments, 4-28.1P, 4-30.1P, 4-31.1P and 4-33.1P, in Region II using the repeat masking program [27] at the website http://www.girinst.org/censor/index.php of the Genetic Information Research Institute. Although transposon or retroviral activities may account for the truncation of other IGHV gene segments (Li et al., unpublished data), no sequence was shown to be transposable element or retrovirus related immediately next to the truncated gene segments in Region II. Therefore, it is likely that these truncations may have been caused by unequal crossovers. The fact that the sequences of paralogous counterparts of the IGHV gene segments in the two complete repeats are very similar and their lengths are either the same or very close indicate that the truncation occurred before the occurrence of duplication in Region II.

### Genetic variation and tag detection

Comparison between the sequences in different assemblies helps us understand why the majority of tags in Region II are polymorphic while a few others are non-polymorphic or undetectable. As shown in Figure 3, sequences in different assemblies share sequence blocks with a high degree of identity. However, these blocks are non-consecutive and interrupted by highly diverged sequences. When a tag is located in a highly diverged area and selected based on a specific sequence assembly, it may not be detectable in a significant portion of samples that do not contain these assembly-specific sequences. It may be detected in all samples if it is located in a highly conserved (or evolutionarily unaffected) region. Conventionally, undetectability of a sequence tag is considered as indication of deletion. However, sequence variants in the polymorphic region presented in the current study suggest that deletion is only one of the possibilities. A high degree of sequence divergence may significantly affect the detectability but there may not be any sequence deletion at all.

### Complexity of Region II

It is possible that more complex duplication/deletion patterns exist in Region II in the human population. Walter et al. [15] described a map with two IGHV gene segments, 3-30 and 4-31 repeated three times (DP49-DP65, DP46-DP78, and DP49-DP65) between the IGHV gene segments 4-28 (DP68) and 4-33 (DP50). On their map, DP64 is an inserting segment between DP46 and DP65 and has a close sequence to 4-31 which is similar to 4-28.1a in the HuRef Assembly in which, however, 4-28.1a is inserted between 4-28.2P and 4-28 (Figure 2). These observations raise two questions: (1) whether 4-31 is located within a highly active genetic element and/or (2) whether the areas at the boundaries of the duplicated units are genetically very active so that exchange in these areas between the duplicated copies occurs at a high frequency. The latter is supported by the results summarized in Table 2. The fact that the sequence tags VHS458, 479 and 504 are not detectable in all haplotypes suggests that sequence divergence in these regions has reached a high level by the frequently occurring genetic events so that tag sequence selected from one haplotype may have significantly diverged from others. On the other hand, although 3-30 and 4-31 were repeated three times and segments 4-28 and 4-33 were also observed in the map described by Walter et al. [15], the remaining eight gene segments observed in the GRCh37 were missing in their map. Such complexity among the haplotypes constitutes a significant source of IGHV diversity in the human population. It also warrants sequence level analysis with haploid materials for thorough and accurate understanding of this region.

### Approach used in the present study to analysis of copy number variants

Copy number variants (CNVs) occupy a significant portion of the human genome [28-33] and may affect the functions of genes [31,34-40] and/or contribute to genetic diseases by altering gene dosage and/or sequences [37,41-44]. So far, large-scale analysis of CNVs has been limited to copy number estimation in diploid genomes. With diploid genomes, it is impossible to learn the number, composition and organization of the CNV copies in individual haplotypes. In addition, the resolution of commonly used microarray-based quantitative methods for studies of copy number changes diminishes as the copy number increases. Our method provides a very efficient way for detailed analysis of CNV regions. With this approach, all known copies of a CNV can be "tagged" so that the number of copy can be counted as "yes" or "no" based on the microarray signals. Using single sperm as subjects, not only the number but also the composition and configuration of the CNV copies in each haploid sample can be determined. We have shown that our multiplex system may be used to analyze more than 2,000 genetic markers in single sperm samples ([2,4], Cui et al., unpublished data). After whole genome amplification of a single sperm, a small aliquot is sufficient for each multiplex amplification reaction. PCR products from many multiplex amplification reactions can be pooled together, allowing thousands of sequences analyzed by a single microarray. Therefore, our system can be easily expanded for high-throughput, comprehensive and detailed CNV analysis.

## Conclusion

Using single sperm as subjects, we identified the haplotype variants in a large region marked by a group of polymorphic DNA sequence tags. Detailed sequence comparison between human genomic sequence assemblies revealed underlying large duplicate sequence blocks. The variants show unusually high level of divergence in their sequences and in IGHV gene segment number, composition and organization, indicating limited selection pressure in general. The underlying mechanism could be one of the major mechanisms responsible for IGHV region diversity. Significant difference between the levels of divergence in the gene segment regions and intergenic region may imply difference in selection pressure on these regions and the functional importance of these IGHV gene segments. Our experimental system with unique sequence tags, single sperm and highly sensitive multiplex DNA sequence detection may be used for study of complex chromosomal regions similar to the IGH region.

## Methods

Briefly, after sperm lysis, the tags were amplified in a single reaction using our high-throughput multiplex PCR amplification procedure [4] with regular primers for all tags. A small aliquot (~2 μl) of the resultant PCR product was then used as template for single-strand DNA (ssDNA) generation using only the N (nested) primers for all tags. Resulting ssDNA was hybridized to the probe sequences arrayed onto a glass slide. Probes were designed in such a way that its 3'-end was next to an A or G base in the ssDNA templates. In the single-base extension assay, two dideoxynucleoside triphosphates, ddUTP and ddCTP conjugated to a fluorescent chromophore (Cy3 or Cy5, respectively) were added so that the chromophores could be incorporated specifically into the probes hybridizing to the templates. The reason for using two dyes instead of one was to monitor the incorporation specificity. Results from microarray analysis are summarized in Table 1.

### Tag sequence selection

Seventeen DNA sequences with an average spacing of ~5 kb were selected based on the sequence published by Matsuda et al. [18] which was used to build the GRCh37 assembly and its earlier versions in the IGH region on chromosome 14. All the DNA sequence tags were chosen from non-coding sequences and designated as "VHS" followed by a number which is the proximal location in "kb" with respect to the first base in the GenBank http://www.ncbi.nlm.nih.gov/Genbank/ sequences of AB019439. The uniqueness of the tags was confirmed by subjecting the sequences containing these tags to the NCBI Blast search program http://www.ncbi.nlm.nih.gov/BLAST/Blast.cgi.

### Design of PCR primers and microarray probes

Three primers were designed for each tag (Table 1). Two primers, forward (F) and reverse (R), were used for multiplex PCR amplification. A nested (N) primer, internal to the F primer in the same direction was designed for generating ssDNA. In addition, a microarray probe (P) internal and close to the nested (N) primer but in the reverse direction, was also designed for each tag. The 3'-ends of the probe sequences were designed next to either an A or G nucleotide in the amplified template sequences so that in the single-base-extension assay (see below), the probes can be labeled by either of the two fluorescently labeled nucleotides, ddUTP or ddCTP. Uniqueness of all the primer and probe sequences was checked using the NCBI Blast search program. Sequences that were not unique were adjusted until they were unique. A primer compatibility test was performed for all the primers by using the software developed in our laboratory [4,45] to avoid primer-primer interactions during multiplex PCR. All oligonucleotides were synthesized by Integrated DNA Technologies, Inc. (Coralville, Iowa). Primer pairs for each tag were tested individually before pooling them together into the multiplex assay. Some primer sequences were reselected as they failed to generate a PCR product with good yield or specificity. All the experiments related to optimizing the conditions for multiplex PCR were performed with 1 ng human genomic DNA before amplifying the tag sequences from single sperm samples.

### Single sperm sample preparation

Semen samples used in the present study were the remains of the specimens used for *in vitro *fertilization and infertility tests. All six donors (002, #12, AB005, AC09, AB027 and D18) were Caucasians and unrelated. They were healthy and normal in fertility. These samples were collected anonymously for a previously unrelated project approved by the Internal Review Board. So, use of these samples should be considered as no human subjects involved according to the US HHS human subjects regulations (45 CFR Part 46).

Sperm cells were purified from semen samples, fixed and stained with propidium iodide [9]. Single sperm were sorted into wells of 96-well V-bottom plates using a fluorescence activated cell sorter. The sorted single sperm samples were lysed in 3 μl of lysis buffer (50 mM dithiothreitol and 200 mM KOH) by incubating at 65°C for 10 min, The lysates were neutralized with 3 μl of neutralization buffer (200 mM HCl, 900 mM Tris-HCl [pH 8.3], and 300 mM KCl) [46]. The samples were then used for multiplex PCR amplification.

### Multiplex PCR amplification

In the first round, all pairs of F and R primers were used to amplify the target sequences from single sperm in a single reaction. Each sample contained 1× PCR buffer (50 mM KCl, 100 mM Tris-HCl, pH 8.3, 1.5 mM MgCl_2, _and 100 μg/ml gelatin), the four dNTPs (200 μM each) (Invitrogen, Carlsbad, California), F and R primers (100 nM each) and 3 units of HotStar *Taq *DNA polymerase (Qiagen, Valencia, California). The final volume for each reaction was 30 μl. The samples were first heated to 94°C for 15 min to activate the *Taq *DNA polymerase followed by 40 PCR cycles. Each PCR cycle consisted of 40 seconds at 94°C for denaturation and 2 minutes at 55°C, followed by 5 minutes of ramping from 55°C to 70°C for annealing and extension. A final extension was carried out at 72°C for 3 min at the end of the 40^th ^cycle. All PCR amplifications were performed with a Biometra T3 Thermocycler (Goettingen, Germany). A small aliquot (2-3 μl) of the first-round PCR product was used as template for generation of ssDNA with only the nested (N) primers. Each sample contained 1× PCR buffer (same as above), four dNTPs (each 100 μM), N primers (50 nM each) and 3 units of *Taq *DNA polymerase with a final volume of 30 μl. The same cycling conditions used in the first round of multiplex PCR were followed.

### Microarray detection of DNA sequence tags in single sperm samples

Gold Seal Micro slides (Becton Dickinson) were soaked in 30% bleach with shaking for 1-2 h, followed by rinsing with MilliQ water. The slides were then sonicated in 15% Fisher brand Versa-Clean liquid concentrate with heat for 1-2 h, rinsed with shaking in MilliQ water and dried by centrifugation at 1000 rpm for 5 min in a Beckman GS-6 centrifuge. It was then baked at 140°C in a vacuum oven for 4-6 h. Each probe was mixed with microarray printing solution for a final concentration of 36 μM in the wells of a 384-well plate. Probes were spotted onto washed glass slides using a microarray spotter, Omnigrid Accent (GeneMachines, San Carlos, California), under a humidity of 50-55% and at a temperature of 22-25°C. The ssDNA was hybridized to the probes in the microarray in 1× hybridization solution (5× Denhardt's solution, 0.5% SDS, and 5× SSC) sealed in a Hybridization Chamber (Corning, NY) at 56°C for 4 h. Before opening, the chambers were immersed in iced water for 30 sec. The slide was then washed in 1× SSC and 0.1% SDS for 10 min, followed by two times in 0.5× SSC for 30 sec, and two times in 0.2× SSC for 30 sec. Probes on the glass slide were then extended by the single base extension assay [46-48]. The assay was carried out using two single dideoxynucleoside triphosphate (ddNTP) (Perkin Elmer, Boston, Massachusetts) conjugated to different fluorescent chromophore (Cy3 or Cy5) using the ssDNA as template. The labeling reaction was done in a solution containing 1/7 volume of Sequenase buffer, 0.5 units/uL Sequenase (Amersham Pharmacia Biosciences, NJ) and 750 nM each of Cy3-ddUTP and Cy5-ddCTP at 70°C for 10 min. After labeling, everything but the labeled probe was washed off as described above. The microarray was then ready for scanning.

Microarrays were scanned with a Genepix 4000B microarray scanner (Axon Instruments, CA). Resulting images were analyzed with the Genepix Pro (Axon Instruments) software. Genotypes were determined by using a computer program developed in our laboratory [4].

### Result validation for the undetectable tags

To further confirm that the undetectable tags were not caused by artifacts, aliquots of the first-round multiplex PCR products from single sperm (9-10 for each tag) of individual AC09 were used for separate re-amplification of undetectable tags using the nested (N) and reverse (R) primers. Two sperm with the detectable tags and tags adjacent to the polymorphic regions were used as positive controls. The amplified products were analyzed by gel electrophoresis. All results were consistent with those from the microarray analysis.

## Authors' contributions

SP designed and carried out a major portion of the experiments, played an important role in data analysis and manuscript preparation. XC and HYW participated in experimental design, data analysis and manuscript preparation. NOC provided very helpful intellectual discussion and manuscript preparation. GH contributed to computer software development for microarray analysis, LS and RG prepared microarrays. HL conceived the study, supervised the entire process, and made substantiate contribution in data analysis and manuscript preparation. All authors read and approved the final manuscript.
